# Hospital and Community Pharmacists’ Perceptions of Which Competences Are Important for Their Practice

**DOI:** 10.3390/pharmacy4020021

**Published:** 2016-06-15

**Authors:** Jeffrey Atkinson, Antonio Sánchez Pozo, Dimitrios Rekkas, Daisy Volmer, Jouni Hirvonen, Borut Bozic, Agnieska Skowron, Constantin Mircioiu, Roxana Sandulovici, Annie Marcincal, Andries Koster, Keith A. Wilson, Chris van Schravendijk, Roberto Frontini, Richard Price, Ian Bates, Kristien De Paepe

**Affiliations:** 1 Pharmacolor Consultants Nancy, 12 rue de Versigny, Villers 54600, France; 2 Faculty of Pharmacy, University of Granada (UGR), Campus Universitario de la Cartuja s/n, Granada 18701, Spain; sanchezpster@gmail.com; 3 School of Pharmacy, National and Kapodistrian University Athens, Panepistimiou 30, Athens 10679, Greece; rekkas@pharm.uoa.gr; 4 Pharmacy Faculty, University of Tartu, Nooruse 1, Tartu 50411, Estonia; daisy.volmer@ut.ee; 5 Pharmacy Faculty, University of Helsinki, Yliopistonkatu 4, P.O. Box 33-4, Helsinki 00014, Finland; jouni.hirvonen@helsinki.fi; 6 Faculty of Pharmacy, University of Ljubljana, Askerceva cesta 7, Ljubljana 1000, Slovenia; Borut.Bozic@ffa.uni-lj.si; 7 Pharmacy Faculty, Jagiellonian University, UL, Golebia 24, Krakow 31-007, Poland; askowron@cm-uj.krakow.pl; 8 Pharmacy Faculty, University of Medicine and Pharmacy “Carol Davila” Bucharest, Dionisie Lupu 37, Bucharest 020021, Romania; constantin.mircioiu@yahoo.com (C.M.); roxana.sandulovici@yahoo.com (R.S.); 9 European Association of Faculties of Pharmacy, Department of Pharmacy, Faculty of Medicine and Surgery, University of Malta, Msida MSD 2080, Malta; annie.marcincal@univ-lille2.fr (A.M.); A.S.Koster@uu.nl (A.K.); 10 Faculty of Pharmacy, Université de Lille 2, Lille 59000, France; 11 Department Pharmaceutical Sciences, Utrecht University, PO Box 80082, Utrecht 3508 TB, The Netherlands; 12 Applied Health Research Unit, School of Life and Health Sciences, Aston University, Birmingham B4 7ET, UK; k.a.wilson@aston.ac.uk; 13 Medical Faculty, Vrije Universiteit Brussel, Laarbeeklaan 103, Brussels 1090, Belgium; chrisvs@vub.ac.be; 14 University Hospital of Leipzig, Centre for Patient Safety, Liebigstrasse 20, Leipzig 04103, Germany; roberto.frontini@medizin.uni-leipzig.de; 15 European Association of Hospital Pharmacists, Rue Abbe Cuypers 3, Brussels 1040, Belgium; richard.price@eahp.eu; 16 School of Pharmacy, University College London, Gower Street, London WC1E 6BT, UK; i.bates@ucl.ac.uk; 17 Pharmacy Faculty, Vrije Universiteit Brussel, Laarbeeklaan 103, Brussels 1090, Belgium; kdepaepe@vub.ac.be

**Keywords:** education, specialisation, practice

## Abstract

The objective of the PHAR-QA (Quality assurance in European pharmacy education and training) project was to investigate how competence-based learning could be applied to a healthcare, sectoral profession such as pharmacy. This is the first study on evaluation of competences from the pharmacists’ perspective using an improved Delphi method with a large number of respondents from all over Europe. This paper looks at the way in which hospital pharmacists rank the fundamental competences for pharmacy practice. European hospital pharmacists (*n* = 152) ranked 68 competences for pharmacy practice of two types (personal and patient care), arranged into 13 clusters. Results were compared to those obtained from community pharmacists (*n* = 258). Generally, hospital and community pharmacists rank competences in a similar way. Nevertheless, differences can be detected. The higher focus of hospital pharmacists on knowledge of the different areas of science as well as on laboratory tests reflects the idea of a hospital pharmacy specialisation. The difference is also visible in the field of drug production. This is a necessary competence in hospitals with requests for drugs for rare diseases, as well as paediatric and oncologic drugs. Hospital pharmacists give entrepreneurship a lower score, but cost-effectiveness a higher one than community pharmacists. This reflects the reality of pharmacy practice where community pharmacists have to act as entrepreneurs, and hospital pharmacists are managers staying within drug budgets. The results are discussed in the light of a “hospital pharmacy” specialisation.

## 1. Introduction

Competence-based learning is not new and is not limited to pharmacy education. The number of published articles on competence-based learning in all areas has risen from one per year in 1982 to 65 per year in 2012 [1]. We investigated how this competence-based approach could be applied to pharmacy. Thus the first objective of the PHAR-QA (Quality assurance in European pharmacy education and training) project [2] was to investigate how competence-based learning could be applied to a healthcare, sectoral profession such as pharmacy in which competences are linked to well-defined outcomes such as patient safety. The second objective of the study concerned the European nature of the pharmacy profession given that pharmacists educated and trained in a given member state have the right, under the freedom of movement directives of the European Union (EU), to practice in another member state [3]. A third objective concerned the organisation of the EU university degree course into a fundamental three-year bachelor course followed by a specialised two-year master course according to the Bologna declaration [4]. Most of the faculties of pharmacy in Europe have a five-year degree course [5]. This is an integrated, seamless model with the pharmacy “bachelor” course at a fundamental level followed by a more specialised “master” course. Here we will examine whether a case can be made for a difference between education and training in future community and hospital pharmacy master degrees.

The discussion on the existence or not of the specialisation of hospital pharmacy has several elements. Regarding work location, graduates with a pharmacy degree are employed in a variety of positions, two of the most important (in terms of numbers) being community and hospital pharmacy (Table 1). Figures for hospital pharmacists vary 10-fold from Spain to the UK. Average figures for employment of pharmacists from the 26 EU member states with university pharmacy departments, published by the PHARMINE “Pharmacy Education in Europe” consortium, were 81% in community and 5% in hospital practice [5]. World-wide figures given by the International Pharmaceutical Federation (FIP) are 55% for community and 18% for hospital practice (with large regional variation) [6]. FIP data for Europe (*n* = 22) is 7.2% of pharmacists working in a hospital, similar to the PHARMINE figure.

Regarding the education for hospital pharmacists, the PHARMINE study [5] reported that university pharmacy departments in 18/26 European countries have a traineeship in a hospital pharmacy during their 5-year course. Thus in the majority of departments the need for training in a hospital environment is recognised as an option.

Regarding the legislation for hospital pharmacists, in some European countries, the status of hospital pharmacist is officially defined by national law and the statutes of the pharmacy professional body, e.g., France, Spain, Italy, Belgium, Netherlands, Portugal, and Switzerland. However, at the European level, this is not a universal approach. In the European Union (EU), the 1985 EU directive on the profession of pharmacy [11] did not recognise any specialisations in pharmacy (although these are recognised in medicine and dentistry). The 2013 update [12] opened up the possibility for specialties of any of the seven automatically recognised professions (medicine, dentistry, veterinary, midwifery, nursing, pharmacy, architects) to be recognised via the creation of a ”Common Training Framework” [13]. A CTF is a new EU tool to achieve automatic professional qualification recognition—including for specialties of pharmacy practice, such as hospital pharmacy—across EU countries.

In the light of the previous chapters, it appears, therefore, that the argument for the existence of a specialised pharmacy job description of “hospital pharmacist” different from that for other specialities such as community pharmacy, although recognised by most university pharmacy departments, lacks a clear common pan-European expression. This is a matter that the European Association of Hospital Pharmacists (EAHP) is seeking to address via a project to form a common training framework for hospital pharmacy in Europe [14].

Pharmacists working in a hospital environment represent a significant sector of practising pharmacists and the hospital pharmacist can be defined by his/her competences and tasks. Differences between hospital and community pharmacists are to be expected in several areas of practice such as patient care and pharmaceutical technology. Community pharmacists are in direct contact with patients and are councillors of ambulatory patients; treatment is frequently symptomatic, based on prescriptions and discussions with the patient, and concerns chronic illness. Hospital pharmacists, and especially clinical pharmacists, are in direct contact with medical doctors and their tasks concern mainly hospitalised patients. Changes in the treatment of hospitalised patients are frequently decided in agreement with the hospital pharmacist. Thus hospital pharmacists are more involved with the treatment starting with interpretation of laboratory tests and diagnosis. The diseases treated are acute and more severe involving complications such as microbial resistance and nosocomial infections that are often evaluated mainly by hospital pharmacists. Hospital pharmacy, however, is evolving in different areas of patient care in the hospital. First, there is a shift towards direct participation in the establishment of treatment by clinical pharmacy teams. The second shift is in the evaluation and establishment of the drug treatment of patients when they leave hospital—an activity similar to that of community pharmacy. Concerning pharmaceutical technology, there has been a shift in medicine production from compounding in pharmacies to industrial production since the 1950s [15]. Compounding in hospital pharmacies has, however, been maintained for paediatric and other specialised formulations.

A conceptual issue here is whether one defines and distinguishes between “community pharmacists” and “hospital pharmacists” on the basis of their working environment or on the basis of different tasks and different responsibilities. Making the distinction on the basis of task/responsibility analysis, as in the case here, will be more useful for curriculum development and for the discussion about differentiated study programmes. Therefore “job description” can be useful, but should not be translated as “working in a hospital.” There are examples of community pharmacists working in hospitals.

Within this context, we investigated in the PHAR-QA (“*Quality Assurance in European PHARmacy Education and Training*”) project [16], whether the ranking by hospital pharmacists of competences for practice is different from that of community pharmacists.

We asked community and hospital pharmacists to rank competences for pharmacy practice. Competences were essentially based on those established in the previous PHARMINE project [17] with input from the MEDINE group [18] who ran a similar project on the evaluation of competences for medical practice by medical doctors and students, and from previous competence frameworks for pharmacy practice [19,20].

This paper describes the similarities and differences between how European hospital and community pharmacists rank competences for pharmacy practice.

## 2. Experimental Section

Ranking data on competences for practice were obtained using the PHAR-QA *surveymonkey* [21] questionnaire that was available online from 14 February 2014 through 1 November 2014 *i.e.*, 8.5 months [22]. Respondents came from 25 EU countries.

The first six questions were on the profile of the respondent (age, duration of practice, country of residence, current occupation (hospital, community, … pharmacist). Questions in clusters 7 through 19 asked about 68 competences for pharmacy practice (see annex). Clusters 7 through 11 were concerned with personal competences, and clusters 12 through 19 with patient care competences. The competences came mainly from PHARMINE [17], MEDINE [18] and the EU directive on sectoral professions [12].

Respondents (hospital pharmacists, *n* = 152, community pharmacists, *n* = 258) were asked to rank the proposals for competences on a four-point Likert scale:

1. Not important = Can be ignored.
2. Quite important = Valuable but not obligatory.
3. Very important = Obligatory, with exceptions depending upon field of pharmacy practice.
4. Essential = Obligatory.

There was also a “cannot rank” possibility, as well as the possibility of leaving the answer blank.

In order to evaluate the effect of age on results, two matched age subgroups were created *post hoc*: age < 40 years old or age > 40 years old. This comparison was based on age rather than duration of practice as (1) in many cases information on the duration of practice was not available (see Table 2); and (2) duration of practice would not take into account late starters.

Results are presented in the form of “scores,” calculated as follows: score = (frequency rank 3 + frequency rank 4) as % of total frequency, *i.e.*, obligatory as a % of total. This calculation is based on that made by the MEDINE consortium in their study on the ranking of competences for medical practice [18]. Such scores are used for descriptive purposes only and no conclusions on statistical differences amongst groups are based on scores.

Leik ordinal consensus [23] was calculated as an indication of the dispersion of the data using an Excel spreadsheet. The original Leik paper cited gives an explicit mathematical example of the calculation of ordinal consensus. Responses for consensus were arbitrarily classified as: < 0.2 poor, 0.21–0.4 fair, 0.41–0.6 moderate, 0.61–0.8 substantial, > 0.81 good, according to the scale proposed in the MEDINE study [18].

The statistical significance of differences amongst groups was estimated from the chi-square test on the original ranking frequencies; a significance level of 5% was chosen. Statistical tests were performed using GraphPad software (Graphpad Software Inc, La Jolla, CA, USA) [24].

Respondents could also add their comments on the different clusters in a text box presented simultaneously with the questions on ranking. An attempt was made to analyse comments using the NVivo10 programme [25] and the Leximancer programme [26] for the semi-quantitative analysis of unstructured data. It was found that the numbers involved were too small to draw significant conclusions, so comments are grouped into clusters.

Ethical concerns on the research included two aspects. First was the avoidance of collecting personal data that was not relevant to the research. The second was to avoid judgement of differences amongst groups. The case of very low ranks for physics and analytical chemistry were treated as misunderstanding following a lack of sufficient clarity in the formulation of the questions. The protocol and results were analysed by the National Commission Drug Bioethics of Romania [27].

## 3. Results and Discussion

The distribution of numbers of respondents by duration of practice of the groups is given in Table 2.

The distributions of duration of practice were not significantly different. In both cases, most respondents had less than 20 years of experience, thus a relatively “young” population seems to be involved.

Respondents in both groups came from 25 European countries. Hospital pharmacy respondents came mainly from Spain (*n* = 36) and the United Kingdom (*n* = 28).

Table 3 shows the overall distribution of rankings by hospital and community pharmacists.

Overall ranking by hospital pharmacists was statistically significantly higher than that by community pharmacists, though the difference was very small.

Only 4.5% (hospital) and 6.9% (community) of respondents were unable to rank competences, suggesting that both groups considered themselves sufficiently informed to reply to the questions asked, and the questions were pertinent to their ideas on practice. This is backed up by the relatively high Leik ordinal consensus values, showing that ordinal dispersion was not great. Thus subgroups do not exist. Similar values for ordinal consensus have been reported by the MEDINE consortium.

**Figure 1:**
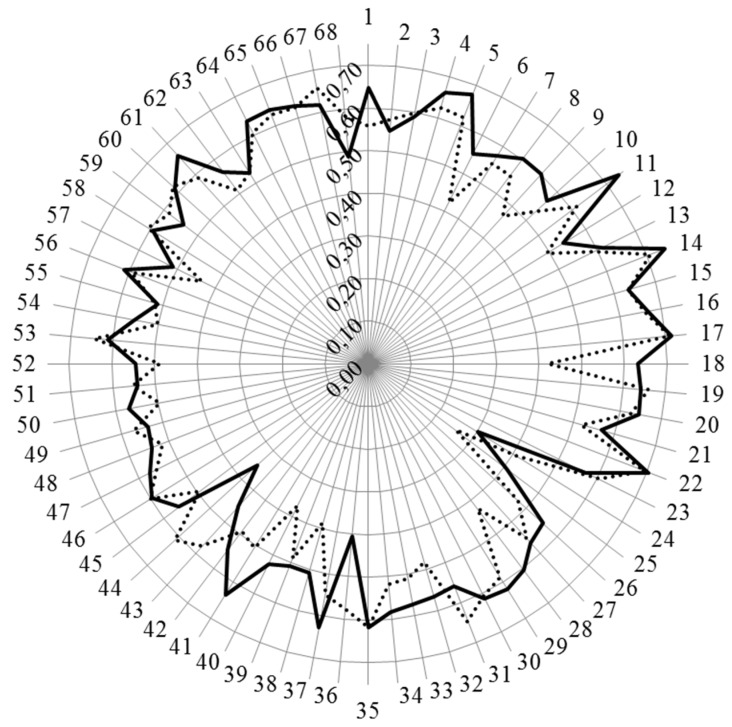
Leik ordinal consensus for rankings by competences for hospital and community pharmacists. Hospital pharmacists: full line; community pharmacists: dotted line.

**Figure 2:**
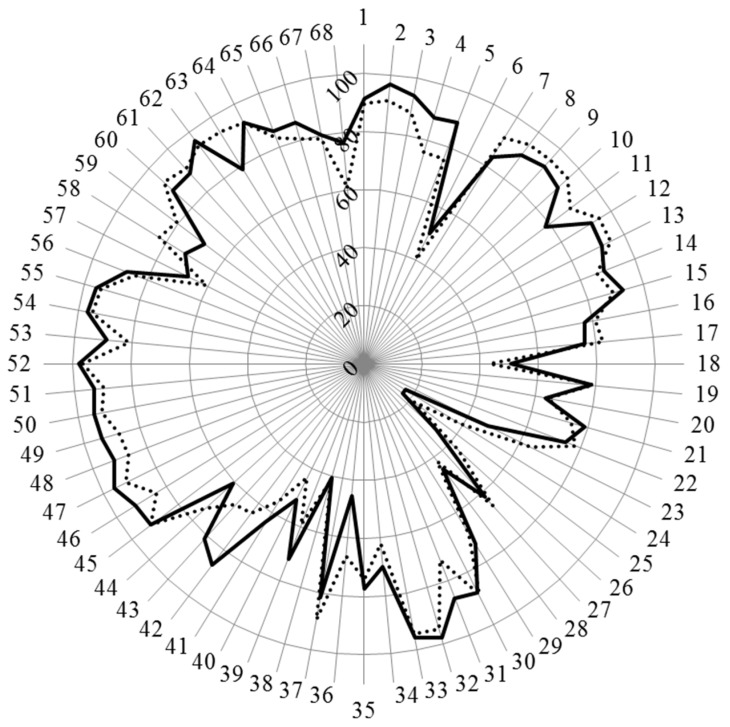
Scores (%) for rankings of competences for hospital and community pharmacists (results in annex). Hospital pharmacists: full line; community pharmacists: dotted line.

Figure 1 and Figure 2 show the results for analysis by competences. Figure 1 shows the values for Leik ordinal consensus.

The ordinal consensus values were in most cases higher than 0.5, and similar in both groups.

Scores for the 68 competences are given in Figure 2 and in detail in table A1 in the appendix.

### 3.1. Cluster 7. Personal Competences: Learning and Knowledge

For the personal competences, cluster 7 “learning and knowledge” (competences 1–7), the scores of hospital pharmacists for competences relating to evaluation and interpretation of, and keeping up to date with, scientific data and evidence-based medical science were higher than those of community pharmacists. Scores for both groups were low for competence 6 related to research but were higher for hospital pharmacists. There was also lower consensus on this competence (Figure 1) showing that, especially for community pharmacists, opinion on the importance of competence in the area of research was split.

There were 32 comments made by hospital pharmacists. These represent ((32/10,336) × 100) = 0.3% of the theoretical total of possible comments. For cluster 7, there were three comments: two on research along the lines of the need for “knowledge = being aware of” rather than “ability = capable of doing.” The third comment expressed sectoral concerns: “*My answers would change if I was looking just at hospital pharmacists or just community pharmacist … I think different skills are of varying importance depending on the sector.*” This concern was expressed elsewhere regarding other competences.

### 3.2. Cluster 8. Personal Competences: Values

For the personal competences, cluster 8 “values” (competences 8–12), the scores of hospital pharmacists were globally lower than those of community pharmacists, especially for those competences concerning human relations such as “inspiring confidence in one’s actions” (competence 11). There were two comments from hospital pharmacists on cluster 8. The first stressed the qualities needed for interaction with other (healthcare) professionals: “*In order to collaborate with other professionals, pharmacists have to show a high degree of responsibility and knowledge.*” The second comment raised the difficulty of evaluating ethical competences: “*I have always wondered how someone can prove 'competence' of approach to human relations and high ethical standards. I would presume that these are not proven…*”

### 3.3. Cluster 9. Personal Competences: Communication and Organisational Skills

For the personal competences, cluster 9 “communication and organisation” values (competences 13–23), the scores of hospital pharmacists were similar to those of community pharmacists except for competence 23 regarding “entrepreneurship,” where hospital pharmacists scored lower than community pharmacists. There were three comments on cluster 9, two expressing sectoral concerns as seen for cluster 7 above. The third concerned language issues: “*Does competency 21 relate to students from English-speaking countries only? Effective communication in the local native language is foremost, important and essential. Good written and spoken English is useful for those wishing to understand research and work at international levels and therefore I'd class this as quite important for non-English speaking countries.*”

### 3.4. Cluster 10. Personal Competences: Knowledge of Different Areas of the Science of Medicines

For the personal competences in cluster 10, “knowledge of the different areas of science” (competences 24–37), the scores of hospital pharmacists were similar to those of community pharmacists except for competences 24 “biology” and 36 “pharmacognosy” where scores for hospital pharmacists were lower, and competence 31 “microbiology” where scores were higher. It should be noted that consensus for competence 24 was low (Figure 1), suggesting that not all agreed on a low score. There were six comments on cluster 10, with one on sectoral concerns as noted above. Three other comments were on the relative importance of the different knowledge areas (greater importance of pharmacology, pharmaceutical technology, *etc.* and of “clinical” subjects”). One comment suggested including social sciences. One comment stressed the need to coordinate tertiary education with secondary education, thereby suggesting that several subjects listed could be dealt with in more detail and depth at the secondary level. It should be noted that cluster 10 was included because considerable emphasis has been placed in the EU directive 2013/55/EU on the recognition of professional qualifications (see above). These are not competencies as generally recognised in the educational literature, syllabus or curriculum. There is no behavioural component, or reflections of values/attitudes which are considered desirable for practice-related competencies. Knowledge of a subject is not a competence in itself but part of a competence. Competencies are generally assumed to comprise behavioural components rather than syllabus components and should be linked to scope of practice. It is the latter which is the important angle, as this is the actual link with patient care. Finally, the educational system may influence these findings, if, for example, a pharmacy student opts for a hospital, community, industry or other track. Thus different educational and training systems may introduce certain biases.

### 3.5. Cluster 11. Personal Competences: Understanding of Industrial Pharmacy

For the personal competences of cluster 11, “industrial pharmacy” (competences 38–42), the scores of hospital pharmacists were higher for four out of five competences relating to legislation and drug production and registration. There were two comments, one again on sectoral concerns. The second concerned the importance of EU directives (competence 40): “*The knowledge about EU legislative is crucial for the pharmacist in the EU countries. For the others—non-EU members—the EU legislative is very important as well, as there is a tendency in unification i.e., maximal improving of the pharmacy practice.*” It is to be noted that the fact that competence 42, “good clinical practice,” is placed within an industrial pharmacy cluster could be interpreted as meaning “good clinical practice in the clinical phase of evaluation of a new chemical entity during the drug registration procedure” rather than “good practice in the exercise of one’s job.” This and other ambiguous phrases were corrected in the second version of the PHAR-QA questionnaire (see the perspective section below).

Ranking of competences in the areas of research, pharmaceutical technology and industrial pharmacy (clusters 10 and 11) are also influenced by the evolution in the way in which medicines are produced. Since the 1950s, several factors have produced a shift in drug production from small-scale, artisanal compounding in pharmacies to large-scale production by the pharmaceutical industry [15]. These factors include the introduction of new drugs, such as antibiotics, vaccines, anti-hypertensives, tranquillizers and antidepressants, the research and development of which requires investment on a large, industrial scale as does the ongoing development of therapies for chronic diseases such as Alzheimer’s disease, the development of biosimilars and the other products of pharmaceutical biotechnology, and the development of treatment with generic drugs [28]. Compounding has continued in specialised hospital settings including the area of sterile preparations and of paediatric therapy [29].

### 3.6. Cluster 12. Patient Care Competences: Patient Consultation and Assessment

For the patient care competences, cluster 12 “consultation and assessment” (competences 43–45), the scores of hospital and community pharmacists were similar except for competence 43 “medical laboratory tests” where hospital pharmacists scored higher. There were six comments on cluster 12, with two on sectoral concerns. Four comments considered that “performing” laboratory tests is not important, whereas interpreting them is: “*Perform tests? I don't think that's relevant with regard to most laboratory tests. Interpretation is of course very important*”.

### 3.7. Cluster 13. Patient Care Competences: Need for Drug Treatment

For the patient care competences of cluster 13 “need for drug treatment” (competences 46–49), the scores of hospital and community pharmacists were similar. There were six comments on cluster 13. Five comments were on the issue of prescribing, e.g.: “*Pharmacists are not allowed to prescribe medicines as they do not know the patient’s clinical background, and drug history*”; “*I think that prescribing would be important—unfortunately a theoretical concern at the moment*”; “*This would depend on the chosen career path following the pharmacy degree. I do not feel it appropriate for a newly qualified pharmacist to prescribe medication due to the lack of clinical experience unless the degree course is significantly extended and leads to specialist qualifications with significant clinical experience similar to doctor's training*”; and “*All pharmacists need to advise doctors but not essential to be able to prescribe.*”

### 3.8. Cluster 14. Patient Care Competences: Drug Interactions

For the patient care competences of cluster 14 “drug interactions” (competences 50–52), the scores of hospital and community pharmacists were similar. There was one comment on cluster 14: “*This should be the expertise of the pharmacist.*”

### 3.9. Cluster 15. Patient Care Competences: Provision of Drug Product

For the patient care competences cluster 15, “provision of drug product” (competences 53–57), the scores of hospital pharmacists were higher for competence 53 “pharmacodynamics and pharmacokinetics” and for competence 57 “ability to manufacture products.” Concerning the latter there was a comment that “*Not sure how most pharmacists would be able to manufacture? Need for license etc.*” It is to be noted that this is another example of possible confusion in that it could be argued that competence 57 belongs in the industrial pharmacy cluster 11.

### 3.10. Cluster 16. Patient Care Competences: Patient Education

For the patient care competences of cluster 16, “patient education” (competences 58–60), the scores of hospital pharmacists were lower than those of community pharmacies. There were no comments on this cluster.

### 3.11. Cluster 17. Patient Care Competences: Provision of Information and Service

For the patient care competences cluster 17, “provision of information and service” (competences 61–63), the scores of hospital and community pharmacists were similar except for competence 63 “non-prescription medicines” where the score of hospital pharmacists (78.9) whilst high, was substantially lower than that of community pharmacists (94.0). There were no comments on this cluster.

### 3.12. Cluster 18. Patient Care Competences: Monitoring of Drug Therapy

For the patient care competences in cluster 18, “monitoring of drug therapy” (competences 64–66), the scores of hospital pharmacists were similar to those of community pharmacies. There was one comment on this cluster: “*I am not sure that pharmacists know current clinical guidelines. If medicine is prescribed we give it to patient.*”

### 3.13. Cluster 19. Patient Care Competences: Evaluation of Outcomes

For the patient care competences cluster 19, “evaluation of outcomes” (competences 67–68), the score of hospital pharmacists was higher regarding the cost effectiveness of treatment (competence 68). There were no comments on this cluster.

Overall the data suggest, firstly, that hospital and community pharmacists have similar ideas on the importance of various competences for practice. Secondly, it would appear that hospital pharmacists rank some of the competences in the context of their own specific activity suggesting that following on from this, certain competences are needed for certain specialisations. Continuing this argument further, after a ground-level competence-based course rooted in, for example, the PHAR-QA framework, certain competences may be necessary in terms of specialisation.

Similar arguments have been put forward for an “industrial pharmacy-oriented” master course given that practice in an industrial environment is very different from that in a community pharmacy environment. In the PHAR-QA survey, we included a cluster on industrial pharmacy; we chose not to include a cluster on hospital pharmacy as we considered that hospital and community practice have many similarities. This is backed up by the results. This does not exclude the fact that some topics such as radio-chemicals, preparation of drugs for specific pathologies, *etc.* are specifically part of hospital pharmacy practice (see later).

**Figure 3:**
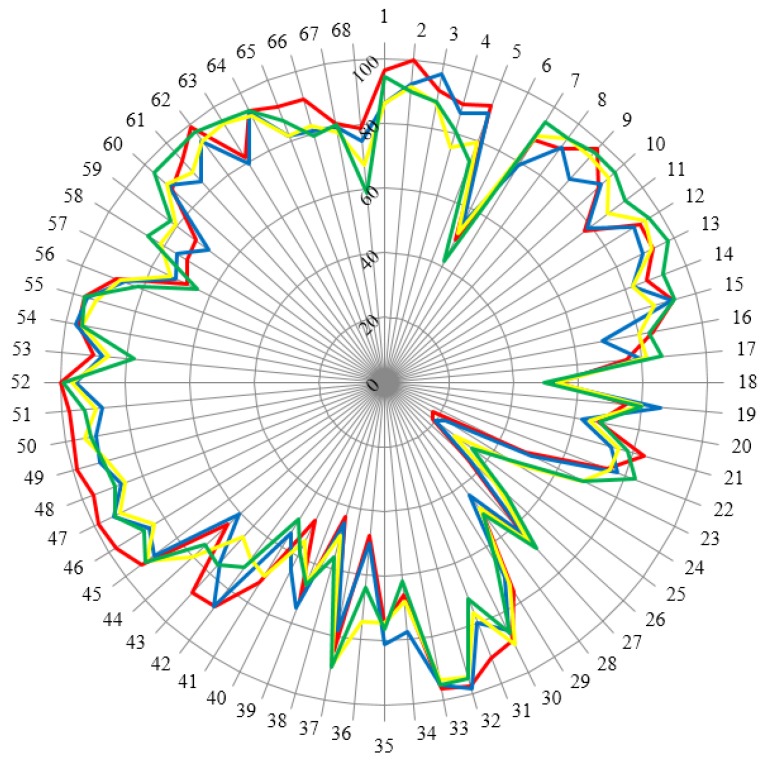
Scores (%) for rankings of competences for hospital and community pharmacists separated into two matched age groups. Hospital pharmacists: < 40 years old: red (*n* = 85), > 40 years old: blue (*n* = 110) Community pharmacists: < 40 years old: yellow (*n* = 148), > 40 years old: green (*n* = 67).

Hospital pharmacists taking part in this survey were not asked for their views on advanced-level competencies and there were few important differences between the hospital and community sectors. This is completely predicated on where these competencies are “located.” Are they “registration" competencies (*i.e.*, applicable to all day 1 pharmacists—in other words a direct outcome of undergraduate/initial education) or are they “foundation” competencies (*i.e.*, related to scope of practice)? To gain further knowledge on this matter, a sub-analysis on the age of the respondents (in terms of two matched age groups) is presented in Figure 3. The duration of practical experience of specialists was considered as a main factor which could induce change and/or refinement of opinions concerning the basic competences required in pharmacy practice. Twenty years was considered as a threshold for change. Such a period is practically a generation. In previous generations, hospital pharmacy was more connected to industrial pharmacy with the preparation of, for example, perfusions for hospital wards and many semisolid formulations. In the same period, community pharmacy involved preparing formulations for short-term use requiring extensive knowledge of chemistry and pharmaceutical technology to avoid problems of stability, *etc.* It was expected that the disappearance of such competences would lead to differences in opinions between younger and older pharmacists. In this context, the duration of practice may be significant. However, as seen in Table 2, the number of responders with more than thirty years’ experience was small (especially community pharmacists), so the effect of “experience” was estimated by effect of “age”.

Figure 3 shows that the group “< 40 years old hospital pharmacists” is somewhat different for many competences especially those in the area of patient care competences. Significantly, there does not appear to be a clear difference between “registration” and “foundation” competences as there are more clear differences between this group and the group of older hospital pharmacists and between this group and age-matched community pharmacists This data would argue for an interaction between age and occupation.

A final consideration concerns the actual value of ranking competencies. One argument used by regulators of health professionals is that registered practitioners are either competent to perform or not. Ranking will imply degrees of competence, and in the eyes of regulators, degrees of patient safety. Furthermore, we can add a cautionary note that statistical difference does not imply an important difference when it comes to difference in a rank order; one example of this is competency 8, “demonstrate a professional approach to tasks and human relations” (90.1% for hospital *versus* 94.5% for community pharmacists), that shows a statistical difference, but represents, independently, a fundamental requirement of scope of practice, wherever you work.

## 4. Conclusions

In general, the agreement on the importance of competencies between different sectors of pharmaceutical activities is high. There was a large consensus between hospital and community pharmacists concerning “patient care,” although the patients are not the same for the two groups. This is shown by the better score for clinical tests given by hospital pharmacists and the greater score given by community pharmacists to prescription drug competences. Other differences can be detected between community and hospital pharmacists. The higher focus of hospital pharmacists on knowledge of the different areas of science as well as on laboratory tests reflects the need of a hospital pharmacy specialisation as supported by the EAHP [14]. The difference is also visible in the field of drug production. This is a necessary competence in hospitals with requests for drugs for rare diseases, paediatric and oncologic drugs. Hospital pharmacists score entrepreneurship lower but cost-effectiveness higher than community pharmacists. This reflects the reality of pharmacy practice where community pharmacists have to act as entrepreneur and hospital pharmacists manage drug budgets. Some results for hospital pharmacists are surprising e.g., the lower score for human relations and patient education. This is probably the consequence of insufficient patient/pharmacist contact in ward rounds in a hospital, and needs some improvement.

This paper provides an innovative contribution to the literature on competence frameworks in pharmacy practice and education. To our knowledge this is the first time a competence framework developed by an academic team has been validated, using Delphi questionnaire methodology, by large populations of different pharmacy practitioners (community, hospital …). It is not the first competence framework for pharmacy practice to be proposed. Several previous frameworks [19,20] have been proposed but these were essentially the results of proposals by a panel of experts without subsequent validation by large numbers of pharmacy practitioners.

Our approach is based on that of the MEDINE group [18] who validated their proposal via a questionnaire sent out to European medical doctors and students. Results were similar to the ones presented here in that the medical community gave high ranking to competences related to general practice (carry out a consultation with a patient, assess clinical presentations, order investigations, make differential diagnoses, and negotiate a management plan, *etc.*). As in our study, the rankings of research topics were low (ability to design research experiments, ability to carry out practical laboratory research procedures, ability to analyse and disseminate experimental results [30], although a second study MEDINE2 suggested that stakeholders considered that “learning outcomes related both to “using research” and “doing research” should be core components of medical curricula” [18]. In contrast to our study, MEDINE looked only at the primary medical degree and did not investigate specialisation.

This paper also represents a first attempt to analyse ranking of competences for community and hospital pharmacy practice and, on the basis of this, to answer the questions: what is a community pharmacist? And what is a hospital pharmacist? Others have considered these questions. In a recent study to determine the use and relevance of the “National Competency Standards Framework for Pharmacists in Australia,” it was found that students, interns and practising pharmacists had poor familiarity and use of the framework [31]. In a study in Thailand, 574 pharmacy practitioners and faculty members ranked pharmacy competency standards. The highest-ranked domain was Domain 1 “Practice Pharmacy within Laws, Professional Standards, and Ethics”; the second and third being Domain 4 “Provide pharmaceutical care” and Domain 3 “Communicate and disseminate knowledge effectively” [32]. Such results are similar to those obtained here.

Other studies have given results different from those in this paper. The Elvey *et al.* [33] study “Who do you Think You Are? Pharmacists’ Perceptions of Their Professional Identity,” a panel of professional pharmacists (*n* = 43, community, hospital, primary care) were asked to give their opinions on the professional identity of the pharmacist. Semi-structured interviews were carried out with questions such as “Describe a pharmacist in five words.” It was interesting that the strongest professional identity to emerge was the “scientist”—but there was considerable overlap with other identities. A somewhat similar conclusion was drawn by Waterfield [34], who asked the question “Is pharmacy a knowledge-based profession?” and found that the importance of the place of science in pharmacy curricula and practice was stressed.

In FIP (2008) [35], for example, several statements have been made regarding the goals and job description of the hospital pharmacy practitioner: “The overarching goal of hospital pharmacists is to optimize patient outcomes through the judicious, safe, efficacious, appropriate, and cost effective use of medicines” and “the ‘five rights’ (the right patient, right medicine, right dose, right route, and right time).” These obviously fit in with the priorities found in this paper as do the conclusions of other think-tank approaches such as that of the Society of Hospital Pharmacists of Australia [36] on general hospital pharmacy practice, and of others on more specialised areas such as clinical research [37]. However, to our knowledge, this is the first paper surveying the opinions of a large number of hospital pharmacy practitioners on their ideas on competences for practice.

## 5. Perspectives

In the light of the rankings and comments, a revised version of the competence framework has been sent out for survey. This will be the basis of the proposal of a PHAR-QA competence framework for pharmacy practice.

The interesting results of the survey support the initiative of the development of a common training framework for a hospital pharmacy post-graduated specialisation as supported by EAHP. The perception of the importance of some specific fields is already shown by the answers of hospital pharmacists. A note of caution is that the conclusions will increasingly be at odds with policy development across EU and wider. Learning from the mistakes made by medical workforce planning, regulators and policy makers now recognise that foundation training (*i.e.*, 2–3 years post-registration) should become the norm (for all sectors) and that advanced “generalism” is a key priority (patient demographics, primary care-based services, *etc.*). In addition, apart from a few competencies discussed here (which are less about “competency” and more about functional tasks—compounding, *etc.*), these outcomes rather suggest a majority similarly in ranking between sectors. The FIP conclusion is that there is significant (both importance and statistical) similarity in foundation competencies [38,39,40].

## Figures and Tables

**Table 1:** Percentages of pharmacists in community and hospital practice in four European countries.

PHARMACISTS	France [7]	Germany [8]	Spain [9]	UK [10]
Community	75	81	58	72
Hospital	12	4	2	23

**Table 2:** Duration of practice (years) in hospital and community pharmacist respondents.

Respondents	Duration of Practice (Years)						
	< 5	6–10	11–20	21–30	31–40	Did not Answer	Total
Hospital pharmacists *n* (%)	37 (24.3)	46 (30.3)	30 (19.8)	26 (17.1)	2 (1.3)	11 (7.2)	152
Community pharmacists *n* (%)	50 (19.4)	51 (19.8)	41 (15.9)	49 (19.0)	7 (2.7)	60 (23.2)	258

*n*: number in each category. Chi-squared test for duration of practice hospital *versus* community = 4.27, degrees of freedom = 4, *p* > 0.05.

**Table 3:** Overall distribution (n = 68 competences) of rankings of hospital and community pharmacists.

RANKING	Hospital Pharmacists		Community Pharmacists	
Number of respondents	152		258	
Theoretical total number of replies	10,336 (=152 × 68)		17,544 (=258 × 68)	
Rank	Number	%	Number	%
4	3948	38.2	6643	37.9
3	3767	36.5	6002	34.2
2	1838	17.8	3076	17.5
1	316	3.1	608	3.5
Cannot rank + blanks	467	4.5	1215	6.9
Score (%)	= ((3948 + 3767)/9869) × 100 = 78.2		= ((6643 + 6002)/16,329) × 100 = 77.4	
Leik ordinal consensus	0.62		0.65	

Chi-square test for comparison of the distribution of ranks for hospital *versus* community pharmacists revealed a significant difference: *p* < 0.05 (degrees of freedom = 3 ((4 ranks − 1) × (2 groups − 1)).

## Appendix

Annex. Ranking of competences by hospital and community pharmacists. (Seq.: sequential numbering (as in figures). Note that the numbering of the clusters of competences starts at 7 ( *i.e.*, continuing from the six questions on the profile of respondents). Competences in bold are those showing a statistically significant difference in distribution of rankings between groups (chi-square, *p* < 0.05).

**Table A1:** Scores (%) for the 68 competences for pharmacy practice as ranked by community and hospital pharmacists.

Cluster	Seq.	Competence	Hospital Pharmacists	Community Pharmacists
Cluster 7. Personal competences: learning and knowledge.	1	Ability to identify learning needs and to learn independently (including continuous professional development (CPD)).	91.4	89.8
	**2**	**Analysis: ability to apply logic to problem solving, evaluating pros and cons and following up on the solution found.**	**96.7**	**91.1**
	3	Synthesis: capacity to gather and critically appraise relevant knowledge and to summarise the key points.	94.0	87.9
	**4**	**Capacity to evaluate scientific data in line with current scientific and technological knowledge.**	**88.0**	**75.8**
	**5**	**Ability to interpret preclinical and clinical evidence-based medical science and apply the knowledge to pharmaceutical practice. **	**89.3**	**75.9**
	**6**	**Ability to design and conduct research using appropriate methodology.**	**50.3**	**40.2**
	7	**Ability to maintain current knowledge of relevant legislation and codes of pharmacy practice.**	**84.0**	**91.7**
Cluster 8. Personal competences: values.	**8**	**Demonstrate a professional approach to tasks and human relations.**	**90.1**	**94.5**
	**9**	**Demonstrate the ability to maintain confidentiality.**	**92.1**	**95.3**
	10	Take full personal responsibility for patient care and other aspects of one’s practice.	90.1	94.8
	**11**	**Inspire confidence in others through actions and advice.**	**78.1**	**88.8**
	**12**	**Demonstrate high ethical standards.**	**92.1**	**95.2**
Cluster 9. Personal competences: communication and organisational skills.	13	Effective communication skills (both orally and written).	91.3	94.8
	14	Effective use of information technology.	88.7	86.1
	15	Ability to work effectively as part of a team.	92.7	89.2
	16	Ability to identify and implement legal and professional requirements relating to employment (e.g., for pharmacy technicians) and to safety in the workplace.	77.2	81.0
	17	Ability to contribute to the learning and training of staff.	76.4	82.5
	18	Ability to design and manage the development processes in the production of medicines.	50.7	43.2
	19	Ability to identify and manage risk and quality of service issues.	78.5	79.2
	20	Ability to identify the need for new services.	63.5	64.5
	21	Ability to communicate in English and/or locally relevant languages.	78.9	74.1
	22	Ability to evaluate issues related to quality of service.	74.3	77.9
	**23**	**Ability to negotiate, understand a business environment and develop entrepreneurship.**	**47.6**	**64.1**
Cluster 10. Personal competences: knowledge of different areas of the science of medicines.	**24**	**Plant and animal biology.**	**16.8**	**39.3**
	25	Physics.	16.8	21.7
	26	General and inorganic chemistry.	33.8	43.9
	27	Organic and medicinal/pharmaceutical chemistry.	61.6	66.0
	28	Analytical chemistry.	45.2	41.9
	29	General and applied biochemistry (medicinal and clinical).	72.8	68.8
	30	Anatomy and physiology; medical terminology.	87.8	88.7
	**31**	**Microbiology.**	**86.4**	**72.2**
	32	Pharmacology including pharmacokinetics.	98.0	94.7
	33	Pharmacotherapy and pharmaco-epidemiology.	95.9	94.3
	34	Pharmaceutical technology including analyses of medicinal products.	70.1	62.0
	35	Toxicology.	77.4	74.0
	**36**	**Pharmacognosy.**	**45.5**	**66.5**
	37	Legislation and professional ethics.	81.6	89.5
Cluster 11. Personal competences: understanding of industrial pharmacy.	38	Current knowledge of design, synthesis, isolation, characterisation and biological evaluation of active substances.	40.7	41.7
	**39**	**Current knowledge of good manufacturing practice (GMP) and of good laboratory practice (GLP).**	**71.9**	**59.4**
	**40**	**Current knowledge of European directives on qualified persons (QPs).**	**52.2**	**43.7**
	**41**	**Current knowledge of drug registration, licensing and marketing.**	**64.0**	**55.7**
	**42**	**Current knowledge of good clinical practice (GCP).**	**86.4**	**64.5**
Cluster 12. Patient care competences: patient consultation and assessment.	**43**	**Ability to perform and interpret medical laboratory tests.**	**81.4**	**65.5**
	44	Ability to perform appropriate diagnostic or physiological tests to inform clinical decision making e.g., measurement of blood pressure.	60.8	73.6
	45	Ability to recognise when referral to another member of the healthcare team is needed because a potential clinical problem is identified (pharmaceutical, medical, psychological or social).	91.8	91.7
Cluster 13. Patient care competences: need for drug treatment.	46	Retrieval and interpretation of relevant information on the patient’s clinical background.	92.3	84.0
	47	Retrieval and interpretation of an accurate and comprehensive drug history if and when required.	95.8	91.5
	48	Identification of non-adherence and implementation of appropriate patient intervention.	92.2	86.8
	49	Ability to advise to physicians and—in some cases—prescribe medication.	93.6	87.6
Cluster 14. Patient care competences: drug interactions.	50	Identification, understanding and prioritisation of drug-drug interactions at a molecular level (e.g., use of codeine with paracetamol).	94.5	91.6
	51	Identification, understanding, and prioritisation of drug-patient interactions, including those that preclude or require the use of a specific drug (e.g., trastuzumab for treatment of breast cancer in women with HER2 overexpression).	93.1	89.7
	52	Identification, understanding, and prioritisation of drug-disease interactions (e.g., NSAIDs in heart failure).	97.9	96.6
Cluster 15. Patient care competences: provision of drug product.	**53**	**Familiarity with the bio-pharmaceutical, pharmacodynamic and pharmacokinetic activity of a substance in the body.**	**88.8**	**81.2**
	54	Supply of appropriate medicines taking into account dose, correct formulation, concentration, administration route and timing.	96.5	94.9
	55	Critical evaluation of the prescription to ensure that it is clinically appropriate and legal.	95.8	94.0
	56	Familiarity with the supply chain of medicines and the ability to ensure timely flow of drug products to the patient.	87.4	84.6
	**57**	**Ability to manufacture medicinal products that are not commercially available.**	**67.6**	**60.5**
Cluster 16. Patient care competences: patient education.	**58**	**Promotion of public health in collaboration with other actors in the healthcare system.**	**72.2**	**82.6**
	**59**	**Provision of appropriate lifestyle advice on smoking, obesity, *etc.***	**68.8**	**80.9**
	**60**	**Provision of appropriate advice on resistance to antibiotics and similar public health issues.**	**88.9**	**93.1**
Cluster 17. Patient care competences: provision of information and service.	61	Ability to use effective consultations to identify the patient’s need for information.	88.7	90.9
	62	Provision of accurate and appropriate information on prescription medicines.	96.5	94.4
	**63**	**Provision of informed support for patients in selection and use of non-prescription medicines for minor ailments (e.g., cough remedies ...).**	**78.9**	**94.0**
Cluster 18. Patient care competences: monitoring of drug therapy.	64	Identification and prioritisation of problems in the management of medicines in a timely manner and with sufficient efficacy to ensure patient safety.	93.0	93.0
	65	Ability to monitor and report to all concerned in a timely manner, and in accordance with current regulatory guidelines on Good Pharmacovigilance Practices (GVPs), Adverse Drug Events and Reactions (ADEs and ADRs).	85.9	83.4
	66	Undertaking of a critical evaluation of prescribed medicines to confirm that current clinical guidelines are appropriately applied.	86.3	80.6
Cluster 19. Patient care competences: evaluation of outcomes.	67	Assessment of outcomes on the monitoring of patient care and follow-up interventions.	80.3	79.0
	**68**	**Evaluation of cost effectiveness of treatment.**	**76.6**	**61.2**

The use of bold text designates competences where ranking frequencies showed a statistically significant difference using the chi-squared test (*p* > 0.05).
